# The Ubiquitin Peptidase UCHL1 Induces G0/G1 Cell Cycle Arrest and Apoptosis Through Stabilizing p53 and Is Frequently Silenced in Breast Cancer

**DOI:** 10.1371/journal.pone.0029783

**Published:** 2012-01-18

**Authors:** Tingxiu Xiang, Lili Li, Xuedong Yin, Chenfu Yuan, Cui Tan, Xianwei Su, Lei Xiong, Thomas C. Putti, Michael Oberst, Kathleen Kelly, Guosheng Ren, Qian Tao

**Affiliations:** 1 Molecular Oncology and Epigenetics Laboratory, The First Affiliated Hospital of Chongqing Medical University, Chongqing, China; 2 Cancer Epigenetics Laboratory, Department of Clinical Oncology, Sir YK Pao Center for Cancer, Li Ka Shing Institute of Health Sciences, CUHK Shenzhen Research Institute, The Chinese University of Hong Kong, Hong Kong, China; 3 Department of Pathology, Yong Loo Lin School of Medicine, National University of Singapore, Singapore; 4 Signal Transduction Section, National Cancer Institute, National Institutes of Health, Bethesda, Maryland, United States of America; The University of Hong Kong, China

## Abstract

**Background:**

Breast cancer (BrCa) is a complex disease driven by aberrant gene alterations and environmental factors. Recent studies reveal that abnormal epigenetic gene regulation also plays an important role in its pathogenesis. *Ubiquitin carboxyl- terminal esterase L1 (UCHL1)* is a tumor suppressor silenced by promoter methylation in multiple cancers, but its role and alterations in breast tumorigenesis remain unclear.

**Methodology/Principal Findings:**

We found that *UCHL1* was frequently downregulated or silenced in breast cancer cell lines and tumor tissues, but readily expressed in normal breast tissues and mammary epithelial cells. Promoter methylation of *UCHL1* was detected in 9 of 10 breast cancer cell lines (90%) and 53 of 66 (80%) primary tumors, but rarely in normal breast tissues, which was statistically correlated with advanced clinical stage and progesterone receptor status. Pharmacologic demethylation reactivated *UCHL1* expression along with concomitant promoter demethylation. Ectopic expression of UCHL1 significantly suppressed the colony formation and proliferation of breast tumor cells, through inducing G0/G1 cell cycle arrest and apoptosis. Subcellular localization study showed that UCHL1 increased cytoplasmic abundance of p53. We further found that UCHL1 induced p53 accumulation and reduced MDM2 protein level, and subsequently upregulated the expression of p21, as well as cleavage of caspase3 and PARP, but not in catalytic mutant *UCHL1 C90S*-expressed cells.

**Conclusions/Significance:**

UCHL1 exerts its tumor suppressive functions by inducing G0/G1cell cycle arrest and apoptosis in breast tumorigenesis, requiring its deubiquitinase activity. Its frequent silencing by promoter CpG methylation may serve as a potential tumor marker for breast cancer.

## Introduction

Breast cancer has been the most common malignancy and the major cause of cancer-related mortality of women worldwide [1]. Although there have been major improvement in the diagnosis and treatment of breast cancer in recent years, early detection methods for breast cancer are still limited. Abnormal promoter methylation is the major mechanism for the inactivation of tumor suppressor genes (TSGs) in tumorigenesis [2], [3]. Increased evidences have demonstrated that aberrant promoter methylation is a promising tumor marker for the early detection of multiple malignancies including breast cancer [4], [5], [6]. Thus, identification of more epigenetically-disrupted TSGs in breast cancer is needed.

Protein ubiquitination plays a critical role in various biological processes including cell proliferation, cell cycle, apoptosis, signal transduction, while its deregulation contributes to tumor initiation and progression [7], [8]. Ubiquitin carboxyl- terminal esterase L1 (UCHL1), a dual-regulator of the ubiquitin proteasome pathway, controls intracellular protein stability by transferring ubiquitin directly to protein substrates and releasing ubiquitin from tandemly conjugated ubiquitin monomers [9], [10]. *UCHL1* has been identified as a cancer-specific methylated gene, and silenced by promoter methylation in multiple tumors including nasopharyngeal (NPC) [11], [12], esophageal squamous cell (ESCC) [13], [14], gastric [15], hepatocellular (HCC) [15], colorectal [15], renal cell [16], and ovarian carcinomas [17]. Our previous work have identified that UCHL1 exerts tumor suppressor activities by deubiquitinating p53 and further activating p53 signaling, thus inhibiting cell proliferation and inducing apoptosis of NPC, HCC and other carcinoma cells [11], . However, the epigenetic disruption of *UCHL1* in breast tumorigenesis and its potential as tumor marker remain ambiguous.

Here, we report that *UCHL1* is frequently methylated in breast cancer cell lines and primary tumors, but rarely in normal breast tissues and mammary epithelial cells, well correlated with its downregulation or silencing. Promoter methylation of *UCHL1* is significantly correlated with pathologic stage of breast cancer and progesterone receptor status. Ectopic UCHL1 expression in breast tumor cells suppresses cell growth, induces G0/G1 arrest and apoptosis through disrupting p53 signaling, depending on its deubiquitinase (DUB) activity, suggesting that UCHL1 is a functional tumor suppressor and potential tumor marker for this cancer.

## Results

### Reduced expression of *UCHL1* in breast cancer

**Figure 1:**
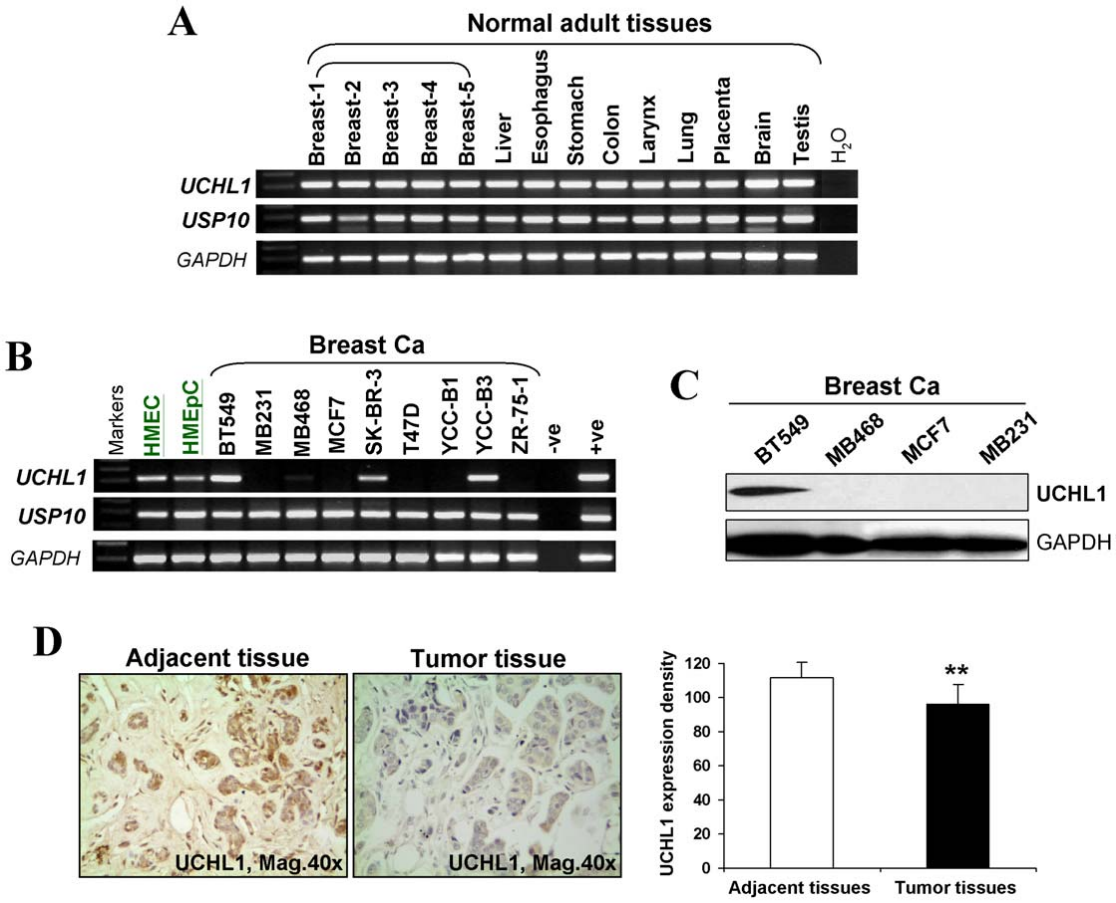
Downregulation of *UCHL1* in breast cancer. (A and B) *UCHL1* and *USP10* expression in normal adult tissues (A), mammary epithelial cell lines and breast cancer cell lines (B), were evaluated using semi-quantitative RT-PCR, with *GAPDH* as a control. (C) UCHL1 protein expression was detected breast cancer cell lines by western blot. (D) Immunohistochemical analysis of UCHL1 in paired adjacent non-cancerous breast tissues and primary breast tumors.

As both UCHL1 and USP10 increase p53 stability by deubiquitination, we first examined the expression of *UCHL1* and *USP10* in normal human tissues including breast tissues, as well as mammary epithelial and breast cancer cell lines, using RT-PCR. *UCHL1* expression was frequently downregulated or silenced in breast cancer cell lines, but broadly expressed in all the normal adult tissues and mammary epithelial cell lines (Fig. 1A and B), while *USP10* is widely expressed in both normal tissues and breast cancer cell lines. Western blot confirmed UCHL1 expression in protein level in breast cancer cell lines, consistent with mRNA level (Fig. 1C).

We further analyzed UCHL1 expression using a tissue microarray carrying 30 breast cancer tissues and paired adjacent non-cancerous tissues by immunohistochemistry. The immunostaining quantification of UCHL1 was analyzed by using Image Pro-Plus (IPP) including 3 parameters: density mean, area sum, and integrated optical density (IOD). Result showed that UCHL1 expression was significantly reduced in breast cancer tissues compared to the adjacent noncancerous tissues (*p*<0.05) (Fig. 1D). These results suggest that *UCHL1* is frequently downregulated in breast cancer.

### Promoter methylation of *UCHL1* and restoration of *UCHL1* expression by demethylation

**Figure 2:**
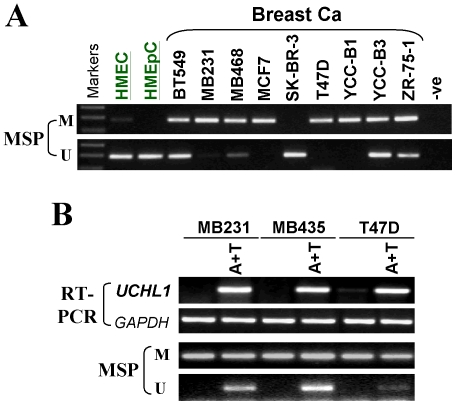
*UCHL1* methylation in breast cancer cell lines and reactivation of *UCHL1* by demethylation. (A) *UCHL1* is frequently methylated in breast cancer cell lines. (B) Pharmacological demethylation restores *UCHL1* expression. A+T: Aza and TSA treatment. M, methylated; U, unmethylated.

We next investigated whether promoter CpG methylation was responsible for the silencing of *UCHL1* in breast cancer. MSP analysis showed that *UCHL1* was frequently methylated in 9/10 (90%) breast cancer cell lines, well correlated with expression levels, while no *UCHL1* methylation was found in two normal mammary epithelial cell lines (Fig. 2A).

We then treated methylated and silenced breast cell lines, MB231, MB435 and T47D, with demethylation reagent 5-aza-2′-deoxycytidine (Aza) and trichostatin A (TSA). RT-PCR showed that *UCHL1* expression was dramatically restored after treatment in these cell lines, together with increase in unmethylated alleles of the *UCHL1* promoter (Fig. 2B). These results demonstrate that promoter methylation of *UCHL1* mediates its silencing in breast cancer.

### *UCHL1* methylation and its clinical correlation in breast tumors

**Figure 3:**
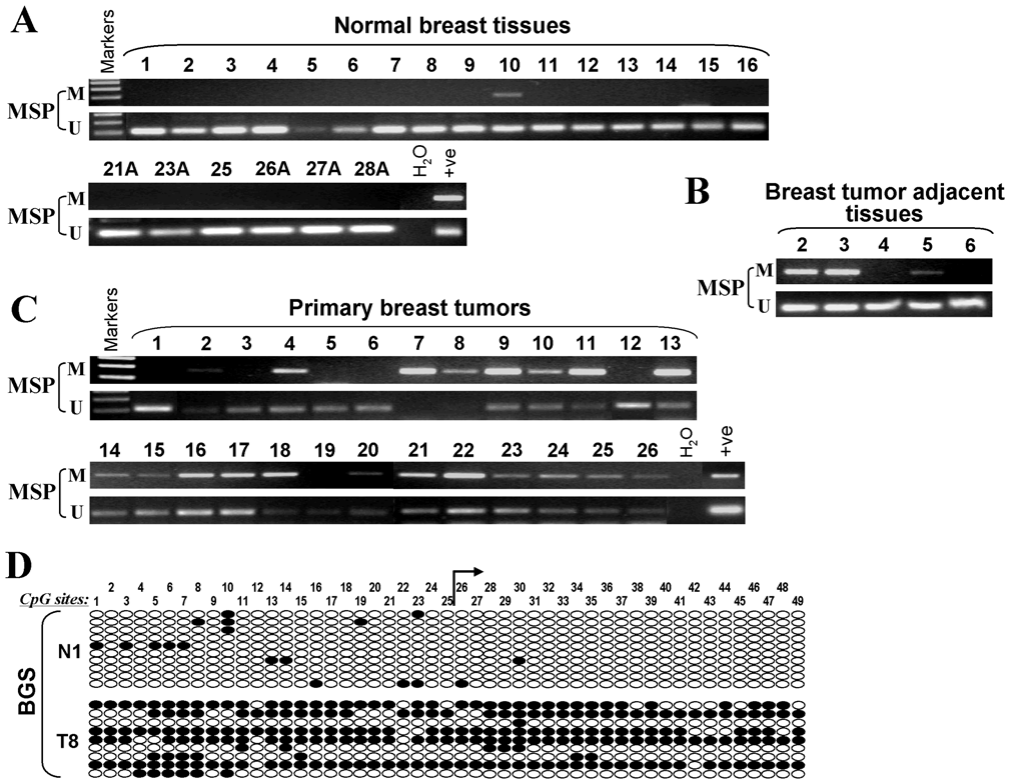
Promoter methylation of *UCHL1* in primary breast tumors. (A, B, C) Representative analysis of *UCHL1* methylation by MSP in normal breast tissues, breast tumor adjacent tissues and primary tumors. M, methylated; U, unmethylated. (D) Detailed BGS analysis of *UCHL1* promoter in primary tumor and normal breast tissue. Circles, single CpG sites analyzed; row of circles, individual promoter allele cloned, randomly selected and sequenced; Filled circle, methylated; empty circle, unmethylated.

To address whether methylation occurs in primary tumors, we analyzed the promoter methylation of *UCHL1* in 66 breast tumor samples, 20 breast tumor adjacent tissues, and 28 normal breast tissues using MSP. *UCHL1* promoter methylation was observed in 53 of 66 (80%) primary tumors, 3 of 20 (15%) adjacent normal tissues and 1 of 28 (3.5%) normal breast tissues (Fig. 3, Table 1). Detailed BGS analysis of representative samples further confirmed the MSP data (Fig. 3D).

**Table 1:** Methylation status of the *UCHL1* promoter in primary breast tumors.

Samples	*UCHL1* promoter		Frequency of methylation
	Methylated	Unmethylated	
BrCa (n = 66)	53	13	53/66 (80%)
BA (n = 20)	3	17	3/20 (15%)
BNP (n = 28)	1	27	1/28 (3.5%)

Note: BrCa, breast cancer; BA, breast cancer adjacent tissues; BNP, breast normal tissues.

We further analyzed the correlation of *UCHL1* methylation with clinicopathological features (Table 2). *UCHL1* methylation was statistically correlated with clinical stage and progesterone receptor (PR) status. However, there was no association of *UCHL1* methylation with other clinicopathological characteristics of patients, including age, histological type, tumor size, lymph node metastasis, oestrogen receptor (ER) and Hormone Receptor (HR) status. These results indicate that promoter methylation of *UCHL1* plays an important role in breast tumorigenesis and might be a potential tumor marker for this cancer.

**Table 2:** Clinicopathological features and *UCHL1* methylation of breast tumors.

Clinicopathological features	Number	*UCHL1* methylation status		*p* value
	(n = 66)	Methylated	Unmethylated	
**Age**				
≤40	5	2	3	*p* = 0.25-0.1
>40	31	24	7	
unknown	30	23	7	
**Stage**				
I	2	0	2	*p*<0.005
II	20	19	1	
III	3	0	3	
unknown	41	30	11	
**Histological type**				
Invasive ductal carcinoma	31	23	8	*p* = 0.75-0.5
Invasive lobular carcinoma	2	2	0	
unknown	33	25	8	
**Tumour size**				
<2.0 cm	18	12	6	*p* = 0.75-0.5
≥2.0 cm≤5.0 cm	18	14	4	
>5.0 cm	0	0	0	
unknown	30	24	6	
**Lymph node metastasis**				
Positive	13	11	2	*p* = 0.75-0.5
Negative	21	16	5	
unknown	32	23	9	
**Oestrogen receptor (ER) status**				
Positive	18	12	6	*p* = 0.75-0.5
Negative	13	11	2	
unknown	35	26	9	
**Progesterone receptor (PR) status**				
Positive	14	8	6	*p*<0.005
Negative	16	11	2	
unknown	30	31	9	
**Hormone Receptor (HR) status**				
Positive	13	9	4	*p* = 0.9-0.75
Negative	17	13	4	
unknown	36	27	9	

### Ectopic UCHL1 expression inhibited colony formation and proliferation of breast cancer cells

**Figure 4:**
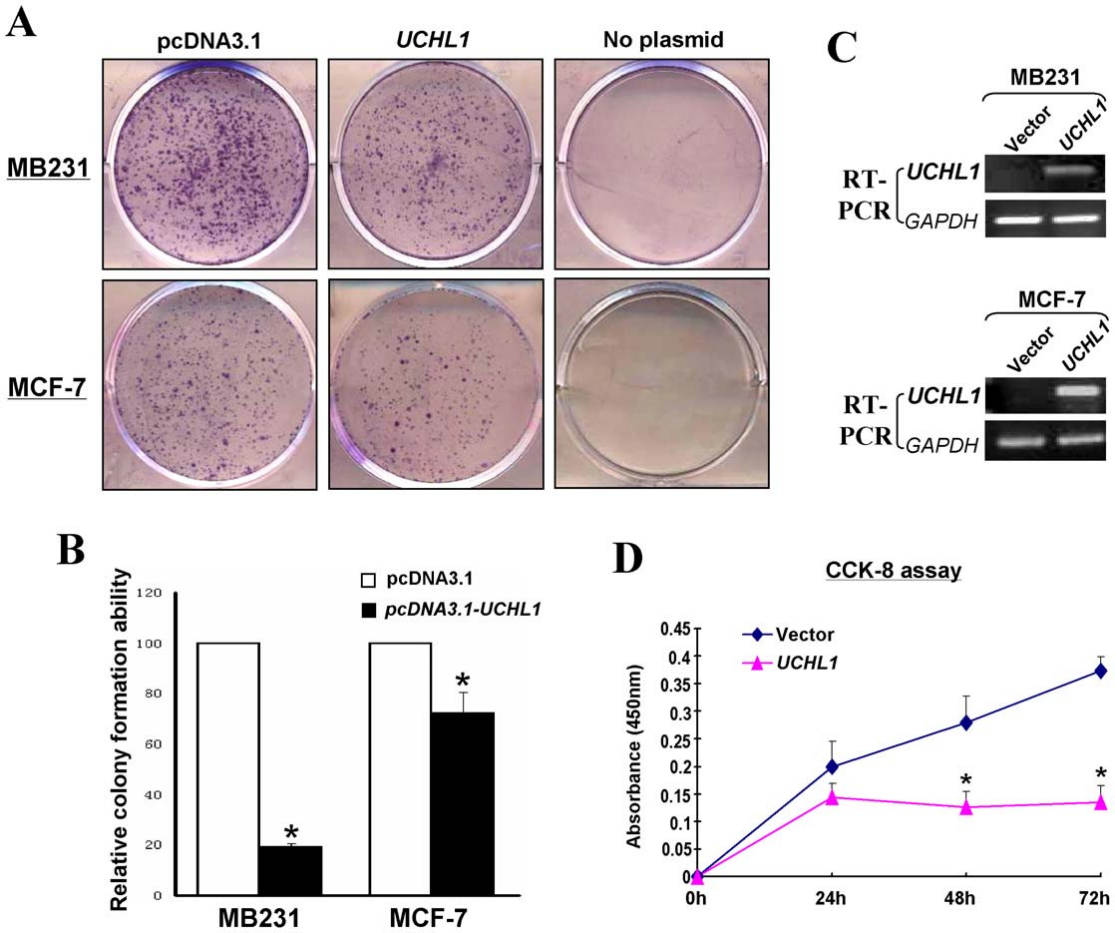
Effects of UCHL1 on the colony formation and cell proliferation of breast cancer cells. (A) Representative colony formation assay. (B) Quantitative analysis of colony formation. The numbers of G418-resistant colonies in vector-transfected controls were set to 100%. Values are expressed as mean±SD from three experiments, and the asterisks indicate statistical significance compared to controls (*p*<0.01). (C) Expression of UCHL1 by RT-PCR in vector- and UCHL1-transfected MB231 and MCF-7 cells. (D) CCK-8 cell proliferation assay for vector- and UCHL1-transfecetd MB231 cells. Asterisks indicate a significant level of proliferation compared to controls (*p*<0.05).

Silencing of *UCHL1* by promoter methylation in breast cancer indicated that *UCHL1* might be a functional tumor suppressor in breast tumorigenesis. We thus performed colony formation assay of MB231 and MCF-7 cells which had complete methylation and silencing of *UCHL1*. Ectopic expression of UCHL1 markedly suppressed the colony formation abilities of breast tumor cells, compared with vector-transfected cells (down to ∼20% and 70%, respectively; Fig. 4). The effects of UCHL1 expression on cell growth at 24, 48 and 72 hrs were also assessed by CCK-8 assay. In *UCHL1*-expressing MB231 cells, cell growth was significantly decreased at 48 h and 72 h (*p*<0.05) (Fig. 4D), but increased in vector-expressing cells in a time-dependent manner. These data suggest that UCHL1 suppresses cell proliferation of breast cancer.

### UCHL1 induced G0/G1cell cycle arrest and apoptosis of breast tumor cells

**Figure 5:**
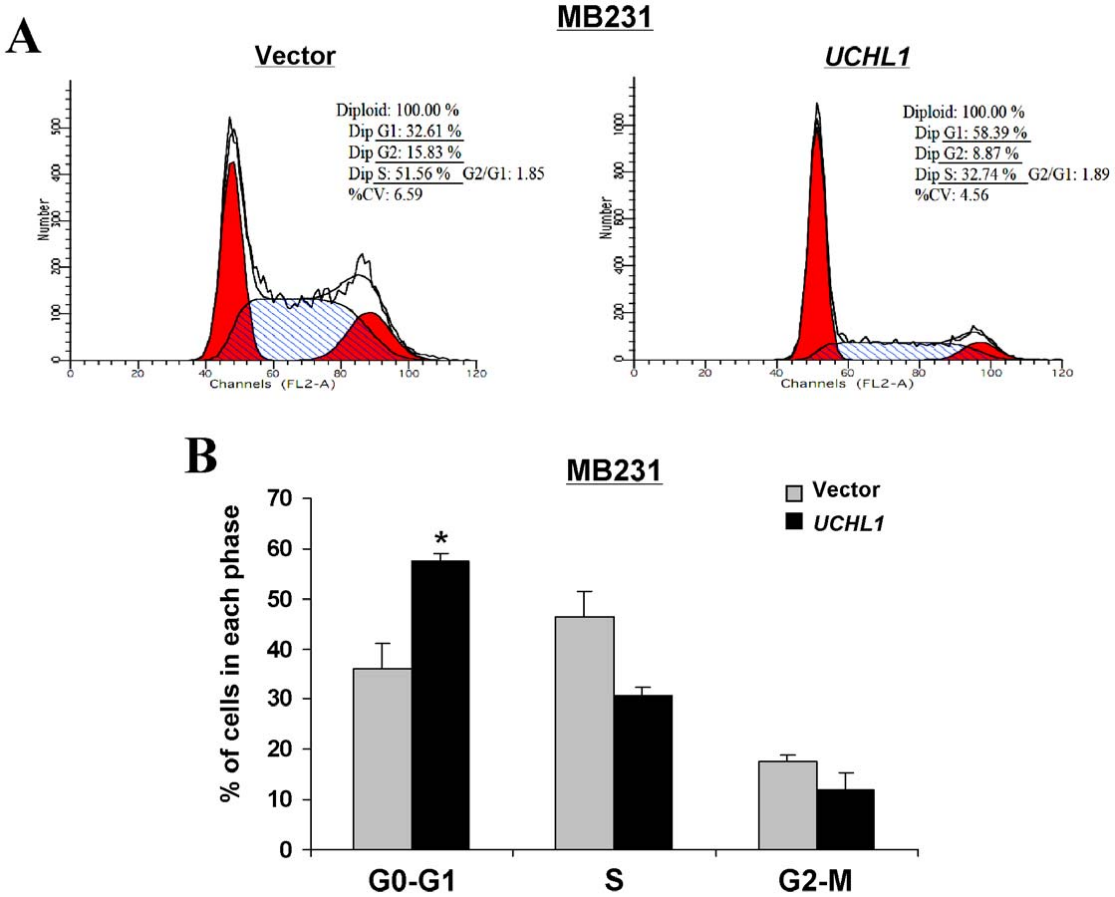
Effect of UCHL1 on cell cycle distribution of MB231 cells. (A) Representative cell cycle analysis. (B) Summarized flow cytometry data. Results are represented as average±S.D and are based on three independent experiments. Statistical significance was determined using Student's t-test (p<0.001).

We further investigated the effects of UCHL1 on cell cycle and apoptosis of breast cancer cells. Representative results of cell-cycle distribution in vector- or *UCHL1*-transfected MB231 cells are shown in Figure 5A. Flow cytometry analysis revealed a statistically significant increase in the number of *UCHL1*-expressing cells with G0/G1phase (21% increase, *p*<0.05; Fig. 5A) accompanied by the decrease of S and G2-M cells, compared to control-transfected cells (Fig. 5B).

**Figure 6:**
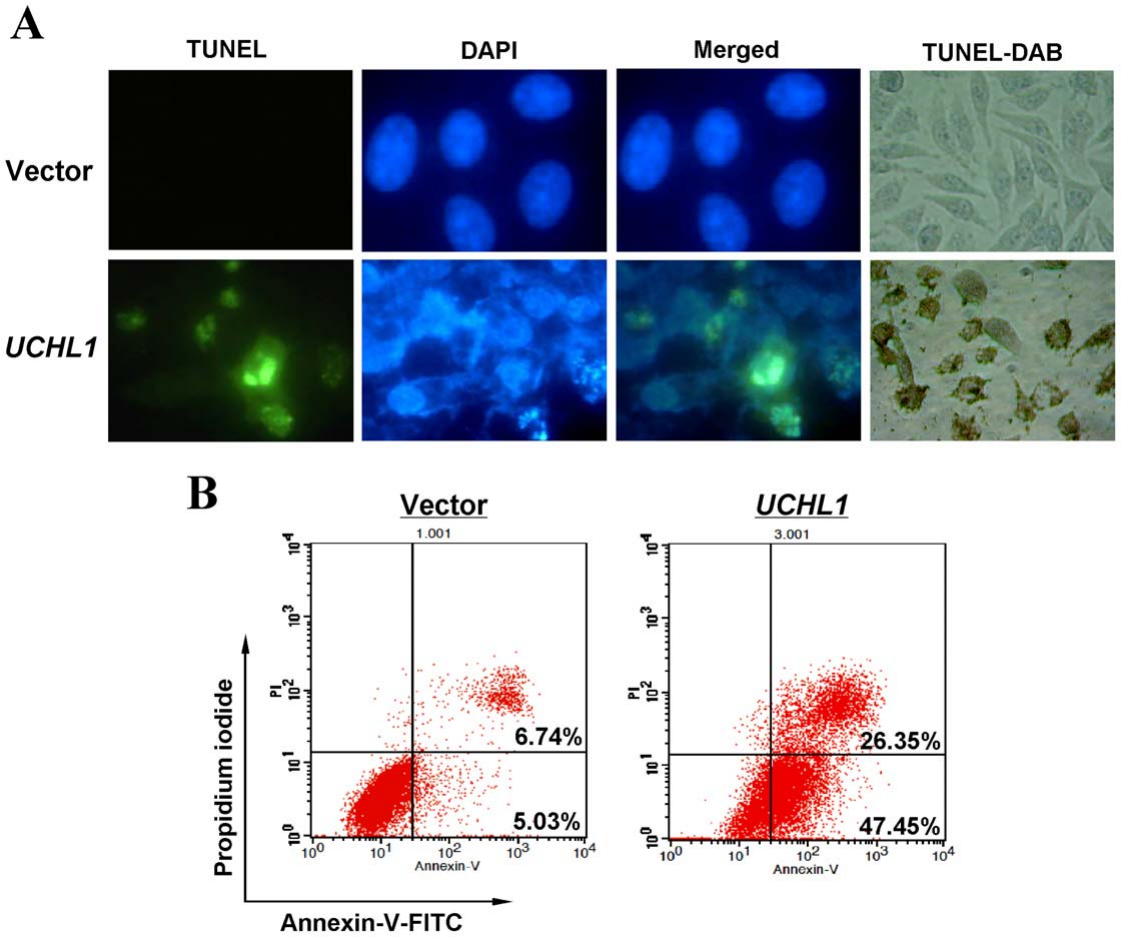
Detection of apoptosis induced by UCHL1 using TUNEL assay and Annexin V-FITC/PI staining in MB231 cells. (A) Apoptotic cells detected by TUNEL staining. (B) Induction of apoptosis detected by flow cytometric analysis with Annexin V-FITC and PI-staining.

Next, we evaluated the apoptotic effect of UCHL1 in breast cancer cells using TUNEL and annexin V-FITC/PI staining assays. TUNEL assay showed that apoptosis was obviously induced in *UCHL1*-transfected MB231 cells (Fig. 6A). Representative Annexin V-FITC staining was shown in Figure 6A. Similar to that measured by the TUNEL assay, the percentage of Annexin V-PI-positive cells was increased in *UCHL1*-transfected MB231 cells and reached 71.8%, compared with controls (Fig. 6B). These results indicate that the inhibitory effect of cell proliferation by UCHL1 is most likely mediated by G0/G1cell cycle arrest and apoptosis.

### Effect of UCHL1 on cell cycle and apoptosis is related to p53 accumulation, requiring its deubiquitinase activity

**Figure 7:**
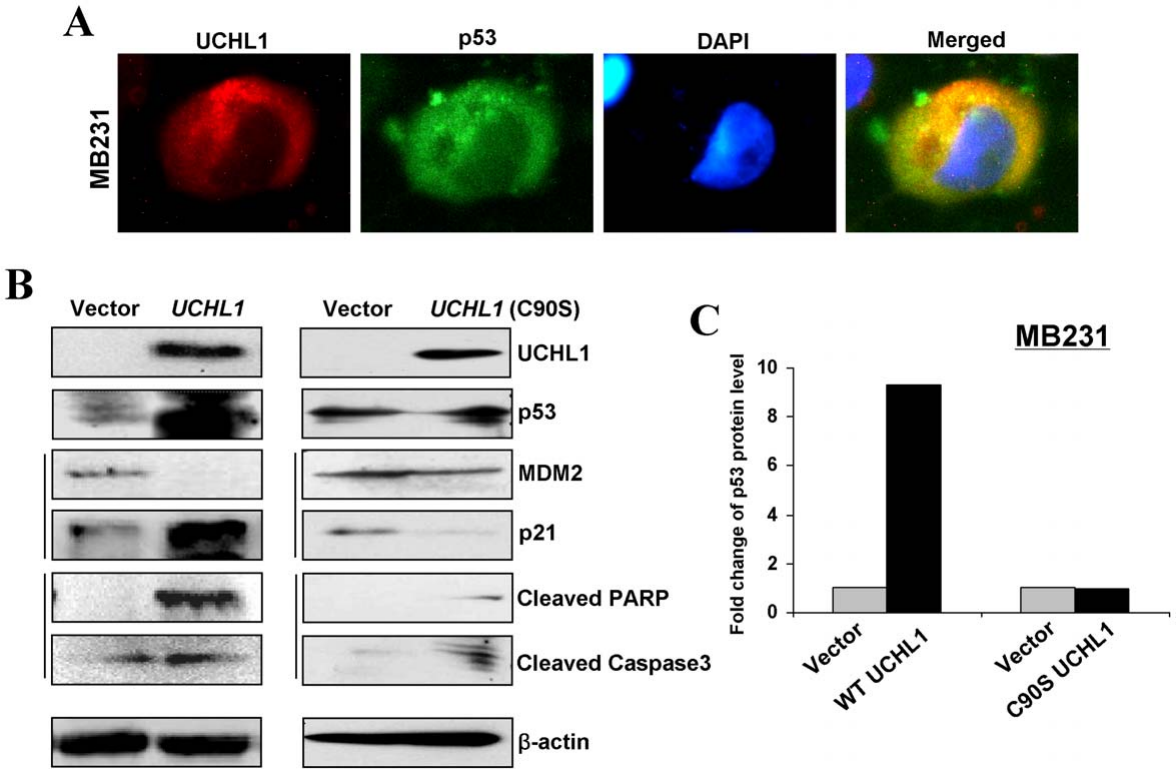
UCHL1 promotes the activation of p53 signaling depending on its deubiquitinase activity. (A) The subcellular localization of UCHL1 and p53 was analyzed in *UCHL1*-transfected MB231 cells. (B) Western blot analysis of cell cycle- and apoptosis-related proteins in vector-, *UCHL1* and *UCHL1 C90S*-transfected MB231 cells. β-actin was used as a control. (C) Quantification of p53 protein expression by Western blot using scanning and ImageJ software. Each sample was normalized to β-actin content.

As both growth arrest and apoptosis are associated with the induction of p53, and UCHL1 has been identified increased p53 accumulation by deubiquitination, we firstly examined the possible correlation between UCHL1 expression and p53 cytoplasmic localization. Immunostaining showed that UCHL1 is primarily located in cytoplasm as expected, and increased the abundance of cytoplasmic p53 in *UCHL1*-expressing MB231 cells (Fig. 7A), but not in the controls (data not shown), suggesting that UCHL1 contributes to cytoplasmic retention of p53.

We then assessed the expression of p53 in *UCHL1*-expressed MB231 cells, using its catalytic mutant UCHL1 C90S as a control in addition to the vector only [10]. Western blot showed that UCHL1 promoted p53 accumulation in breast tumor cells, along with the reduction of MDM2, while UCHL1 C90S did not increase p53 accumulation, but partly decreased the expression of MDM2 (Fig. 7B). In MB231- expressing UCHL1 cells, p53 protein level was showed 9-fold or greater changes compared to the controls, but no any change was observed in *UCHL1 C90S*- expressing cells (Fig. 7C). We further found the expression of p21 was dramatically increased in *UCHL1*-expressing cells, which are the key G1/S cell cycle regulators and p53 downstream target genes, as well as cleaved-caspase 3 and PARP (Fig. 7B), while little or no upregulation of these markers was observed in *UCHL1 C90S*- expressing cells. Thus, upregulated p53-signaling by UCHL1 through its DUB activity was involved in G0/G1 cell cycle arrest and apoptosis in breast cancer cells.

## Discussion

In the present study, we analyzed the epigenetic alteration of *UCHL1*, its tumor suppressive functions and related-mechanisms in breast cancer. We found that *UCHL1* was abundantly expressed in normal breast tissues and normal mammary epithelial cell lines, but frequently downregulated or silenced in breast cancer cell lines and primary tumors due to its promoter methylation, indicating that aberrant promoter methylation is a major cause for *UCHL1* disruption in breast cancer. In primary tumors, *UCHL1* methylation was associated with clinical stage and progesterone receptor status, indicating its potential as tumor marker for this cancer. Functionally, ectopic expression of *UCHL1* suppressed the colony formation and cell proliferation of breast tumor cells through inducing G0/G1cell cycle arrest and apoptosis. Furthermore, we found that the p53 accumulation is mainly due to its cytoplasmic retention by *UCHL1*, depending on its DUB activity, which is responsible for the tumor suppressive function of *UCHL1*. Thus, *UCHL1*, acts as a functional tumor suppressor by inhibiting cell proliferation and inducing apoptosis, but is epigenetically-silenced in breast cancer.

Abnormal of ubiquitin-proteasome signaling pathway is closely associated with multiple tumorigenesis, including breast cancer [8]. Malfunctions in the ubiquitin- proteasome system enhance the effects of oncoproteins, reduce the protein levels of tumor suppressor proteins, further leading to the inhibition of apoptosis of tumor cells and promotion of cell proliferation [18]. *UCHL1*, located at 4p14, was first reported as a member of the ubiquitin proteasome pathway [19], and plays an important role in controlling intracellular ubiquitin levels in cells undergoing ubiquitin-dependent protein degradation [9], [20]. Promoter methylation has been identified to be the major cause for *UCHL1* downregulation or silencing in multiple malignancies. Although high expression of UCHL1 was reported to predict early recurrence in patients with invasive breast cancer [21], other evidences indicated the potential of UCHL1 as tumor suppressor in breast cancer. Overexpression of UCHL1 has been found to induce apoptosis in MCF-7 cells [22]. Using genomic screening upregulated genes by demethylation agent treatment in breast cancer cells, Fujikane et al found that *UCHL1* was methylated in primary breast tumors [23], consistent with our studies.

Our previous work demonstrated that UCHL1 could activate the p14ARF-p53 signaling pathway by deubiquitinating p53 and p14ARF as well as ubiquitinating MDM2, which might be through its two opposing enzyme activities, hydrolase and ligase, further resulting in its tumor suppressive role in NPC tumorigenesis [11], [15]. Although USP10 has been reported to regulate p53 localization and stability by deubiquitinating p53 directly [24], our data showed that *USP10*, unlike *UCHL1*, is widely expressed in both normal tissues and breast cancer cell lines, and no any expression correlation in mRNA level between *USP10* and *UCHL1* was found in breast cancer, indicating UCHL1 is mainly responsible for p53 stability in breast cancer.

UCHL1 is primarily located in cytoplasm exerting its ubiquitinase fucntion, and p53 is also reported to be localized in cytoplasm in quiescent mammary gland without hormone treatment, which is not responsible for p21 transcription [25]. Another report suggested that cytoplasmic p53 was usually wide type and detected in normal breast tissue while mutated p53 is located in nucleus in breast cancer tissues [26]. In this study, we found that UCHL1 retained p53 in the cytoplasm substantially. Furthermore, using catalytic mutant UCHL1 C90S as a control, the accumulation of p53 mediated by UCHL1 was observed, subsequently, p21, as key p53 downstream target genes and regulators of cell cycle G1/S checkpoint, as well as cleaved-caspase 3 and PARP, were obviously upregulated, accompanied by UCHL1-mediated p53 activation, but not in *UCHL1 C90S*-expressing breast cancer cells. We also found a dramatic reduction of MDM2 in *UCHL1*-expressing cells, but minor change was observed in *UCHL1 C90S*-transfected cells, which is well correlated with the function of UCHL1 C90S, lacking hydrolase activity but maintaining binding affinity for ubiquitin. However, the study on the mechanism of the negative correlation between UCHL1 and MDM2 level needs to further investigated. Thus, ensuring proper p53 signaling is tightly related to UCHL1-induced tumor suppression in breast pathogenesis.

Recent studies have shown that *UCHL1* methylation is correlated with tumor cell differentiation, lymph node metastasis and poor prognosis, thus as a tumor marker [12], [13], [23], [27], [28]. *UCHL1* promoter methylation is an independent prognostic factor for ESCC survival and thus a valuable tumor marker for ESCC progression [13]. We also identified that *UCHL1* methylation as a biomarker for HCC and other digestive tumors previously [15]. In breast cancer, we found that the promoter methylation of *UCHL1* was a promising marker indicative of breast cancer progression.

In summary, we found that UCHL1 possesses tumor-suppressive functions in breast tumor cells requiring its DUB activity, and is frequently silenced by promoter methylation, thus as a potential tumor marker for breast cancer. Our study further extends the current understanding of the role of epigenetically-disrupted tumor suppressor gene in breast tumorigenesis.

## Materials and Methods

### Cell lines and tumor samples

Ten breast tumor cell lines (BT549, MB435, MCF-7, T47D, MDA-MB-231, MDA-MB-468, ZR75-1, SK-BR-3, YCC-B1 and YYC-B3) were used [29], [30]. Human normal mammary epithelial cell lines HMEpC and HMEC (CA-830-05a, Applied Biosystems, Foster City, CA) were used as controls. All cell lines were maintained in RPMI 1640 (Gibco-BRL, Karlsruhe, Germany) supplemented with 10% fetal bovine serum (FBS) (PAA Laboratories, Linz, Austria), 100 U/ml penicillin and 100 µg/ml streptomycin, at 37°C in a humidified atmosphere containing 5% CO_2_ [29], [30], [31]. RNA samples of human normal adult tissues were purchased commercially (Stratagene, La Jolla, CA; Millipore Chemicon, Billerica, MA and BioChain Institute, Hayward, CA). DNA samples of breast tumor, adjacent tissues and normal breast tissues have been described previously [32], [33].

Some fresh breast tumors, adjacent non-cancerous tissues and normal breast tissues were obtained from patients treated by primary surgery at the First Affiliated Hospital Surgery Department of Chongqing Medical University (Chongqing, China). All samples were evaluated and subjected to histological diagnosis by expert pathologists. Grading of tumors was achieved by staining with hematoxylin and eosin (H&E). The clinical data, including race, age, site of primary tumor, stage, receptor status and tumor differentiation, were also obtained. All patients provided informed consent for the study to retain and analyze their tissues for research purposes. The samples were immediately snap-frozen following resection and stored in liquid nitrogen until processing. The study was approved by Institutional Review Board of the Chongqing Medical University. The written informed consent was obtained.

### DNA and RNA preparation

DNA and RNA were isolated as previously described [34], [35]. Briefly, RNA was extracted using TRIzol reagent (Life Technologies Inc., Carlsbad, CA). DNA was extracted using Qiagen DNeasy Tissue kit (Qiagen, Inc., Valencia, CA). Extracted DNA and RNA were quantified using spectrophotometry analysis. Samples were stored at −20°C or −80°C until use.

### Semi-quantitative RT-PCR

Semi-quantitative RT-PCR of *UCHL1* using Go-Taq (Promega, Madison, WI) was performed as previously described with GAPDH as a control [11], [15], [34]. Primers used were *UCHL1*F: 5′-AGCTCAAGCCGATGGAGATC; *UCHL1*R: 5′-CCCTTCAGCTCTTCAATCTG, *GAPDH*F: 5′-TCCTGTGGCATCCACGAAACT; *GAPDH*R: 5′-GAAGCATTTGCGGTGGACGAT. RT-PCR was done with 32 cycles for UCHL1 and 23 cycles for GAPDH.

### Tissue microarray and immunohistochemistry

To evaluate the expression levels of UCHL1 protein in breast cancer tissues, tissue microarray (TMA) was constructed using paraffin-embedded, formalin-fixed tissues from 31 patients, including primary tumor and adjacent tissues, and one pair as positive controls (Biochip Co., Ltd., Shanghai, China). Immunohistochemistry was performed using UCHL1 polyclonal antibody (ab10404, Abcam). Briefly, sections were deparaffinized, subjected to microwave antigen retrieval for 15 min in sodium citrate solution (pH 6.0) and then incubated with 3% hydrogen peroxide to block endogenous peroxidase activity. The sections were incubated with primary antibody (1∶1000 dilution) overnight at 4°C, followed by second antibody (1∶2000 dilution) at 37°C for one hour. Finally, the slides were counterstained with hematoxylin. Negative control was performed by replacing the primary antibody with PBS.

Evaluation of UCHL1 expression by IPP (version 6.0, Media Cybernetics, Silver Spring, MD) as described previously [36]. Briefly, 5 digital images at 1360×1024 pixel resolution at 400 magnification were captured by the LEICA DM500 ICC50 microscope (Leica microsystems, Germany).The measurement parameters included density mean, area sum, and IOD. The optical density was calibrated and the area of interest was set through: hue, 0∼30; saturation, 0∼255; intensity, 0∼255, then the image was converted to gray scale image, and the values were counted.

### Methylation-specific PCR (MSP) and bisulfite genomic sequencing (BGS)

Bisulfite modification of DNA, MSP and BGS were performed as described previously [37], [38]. The bisulfite-treated DNA was amplified with the methylation-specific primer set *UCHL1*-m1: 5′-TTTATTTGGTCGCGATCGTTC ,*UCHL1*-m2: 5′-AAACTACATCTTCGCGAAACG or the unmethylation specific primer set *UCHL1*-u1: 5′-GGGTTTGTATTTATTTGGTTGT, *UCHL1*-u2: 5′-CTTAAACTACATCTTCACAAAACA. MSP was done using AmpliTaq Gold (methylation-specific primers: annealing temperature 60°C, 40 cycles; unmethylation- specific primers: annealing temperature 58°C, 40 cycles).

For BGS, bisulfite-treated DNA was amplified using primers *UCHL1*-BGS1: 5′-GTTTTATATATTTAAGGAATATTT and *UCHL1*-BGS2:

5′-CTTAATCCCTACC AACAAC. The PCR products were cloned into pCR4-Topo vector (Invitrogen, CA), with 8 to 10 colonies randomly chosen and sequenced.

### Flow cytometry analysis of cell cycle and apoptosis

Flow cytometry analyses of cell cycle and apoptosis were described previously [29], [39]. For cell cycle analysis, cells were fixed in ice-cold 70% ethanol and stained with propidium iodide (PI). The cell-cycle profiles were assayed using the Elite ESP flow cytometry at 488 nm, and the data were analyzed using the CELL Quest software (BD Biosciences, San Jose, CA). For apoptosis analysis, Annexin V-FITC/PI staining was also performed using flow cytometry according to the manufacturer's guidelines. Briefly, cells were incubated with PI and Annexin V-fluorescein isothiocynate in the darkness at room temperature. Flow cytometric analysis was immediately performed.

### Colony formation assay

For monolayer culture, freshly seeded tumor cells (2×10^5^/well) were plated in a 6-well plate, cultured overnight, and transfected with pcDNA3.1-UCHL1 or pcDNA3.1 plasmid using Lipofectamine 2000 (Invitrogen, CA). Cells were plated in a new 6-well plate 48 h post-transfection, and selected for 1–2 weeks using G418 (1200 µg/ml), while untransfected cells would not survive G418 selection. Surviving colonies (≥50 cells per colony) were counted after staining with Giemsa14 days post-transfection, counted and photographed. Each experiment was run in triplicate, and performed for three times.

### Cell proliferation assay

For the analysis of cell growth rate, MB231 cells transfected as above and plated in 96-well plate at a density of 5×10^3^ cells/well, and cell proliferation assay was measured at 24, 48 and 72 h using the Cell Counting Kit-8 (CCK-8) (Dojindo Molecular Technologies, Japan) according to the company's instruction. Spectrometric absorbance at 490 nm was measured using a microplate reader. The data were obtained from three independent cultures and each experiment was repeated four times.

### TUNEL assay

Analysis of apoptotic cells was performed using the terminal deoxynucleotidyl transferase-mediated dUTP nick-end labeling (TUNEL) staining kit following the manufacturer's instruction (Roche, CA). Briefly, MB231 cells were grown on glass coverslips and transfected with pcDNA3.1-UCHL1 or pcDNA3.1 plasmid and cultured for 48 h. Transfected cells were then washed with PBS, fixed with 4% paraformaldehyde in PBS, and incubated with primary antibodies. The nuclei were counterstained with DAPI (Roche, CA). TUNEL-positive cells had a pyknotic nucleus with dark green fluorescent staining, indicative of apoptosis. The TUNEL reaction was also visualized by chromogenic staining with DAB (002114, Invitrogen, CA). Images of the sections were taken using a fluorescence microscope (Leica DM IRB).

### Indirect Immunofluorescence

Cells grown on coverslips were stained by indirect immunofluorescence as described previously [39]. Briefly, cells were incubated with primary antibodies against UCHL1 and p53, and then incubated with Alexa Fluor 555-(Invitrogen Molecular Probes, Carlsbad, CA) or FITC-conjugated (Dako, Denmark) secondary antibody against mouse or rabbit Ig G. Cells were then counterstained with DAPI and imaged with a fluorescence microscope (Leica DM IRB).

### Construction of UCHL1 C90S expression vector

UCH L1 C90S mutant was introduced by PCR-based site-directed mutagenesis (QuickChange Site-Directed Mutagenesis Kit, Stratagene, La Jolla, CA, USA) of pcDNA3.1-UCHL1 plasmid using primers designed to introduce specific mutations (C90S) and sequence verified.

### Western blot

Western blot analysis was performed as described previously [11]. The primary antibodies were used: UCHL1 (ab10404, Abcam), cleaved caspase-3 (9661, Cell Signaling Technology, Danvers, MA), cleaved PARP (9541,,Cell Signaling Technology, Danvers, MA), p53 (sc-126, Santa Cruz, CA), MDM2 (sc-813, Santa Cruz, CA), p21/Cip1 (2990-1, Epitomics, USA), and β-catenin (1247-1, Epitomics, Burlingame, CA). p53 protein level was determined and quantified by western blot using the ImageJ.

### Statistical analysis

Statistical analyses were performed using SPSS version 16 software. The expression analysis of UCHL1 between BrCa tissues and the corresponding adjacent tissues were assessed using the Student's t-test. Comparisons of categorical variables were performed using the χ^2^ test or 2-tailed t-test. Fisher's exact test was used when appropriate. Differences were considered statistically significant if a *p* value was less than 0.05.
